# Identification of potential biomarkers for osteoporosis and chronic kidney disease through bioinformatics and machine learning algorithm

**DOI:** 10.1371/journal.pone.0348515

**Published:** 2026-05-04

**Authors:** Hao Tang, Kai Hu, Yuankang He, Zihao Zhang, Bingcheng Liu, Xiao Ma, Tianwen Ye

**Affiliations:** 1 Department of Orthopaedic Surgery, Changzheng Hospital, Naval Medical University, Shanghai, China; 2 Basic School of Medicine, Naval Medical University, Shanghai, China; University of Vermont, UNITED STATES OF AMERICA

## Abstract

**Objective:**

The purpose of this study is to identify hub genes associated with both osteoporosis (OP) and chronic kidney disease (CKD) through bioinformatics analysis, and to explore the potential pathogenetic mechanisms in OP and CKD through these hub genes.

**Methods:**

We downloaded the GSE15072 and GSE56815 datasets from the GEO database as training sets, and GSE7158 and GSE70528 for validation. Differential expression genes were selected using the “limma” package, while gene co-expression networks were constructed with “WGCNA.” Functional enrichment analyses were performed using “clusterProfiler.” Hub genes were identified through machine learning techniques, and their diagnostic efficacy was evaluated by ROC curves plotted with the ‘pROC’ package. Immune infiltration was analyzed using CIBERSORT, and pan-cancer relationships were explored to identify associations between hub genes and various tumors. Potential therapeutic agents were investigated using the Drug Signatures Database (DSigDB). Experimental validation was conducted via RT-qPCR using cisplatin-induced chronic kidney disease (CKD) and ovariectomy (OVX)-induced osteoporosis models in C57BL/6J mice. After anesthesia and sacrifice, peripheral blood mononuclear cells (PBMCs) were collected to analyze the expression changes of hub genes.

**Results:**

This study identified four hub genes (FAM184A, NFKBIA, RP2, HIRA). All hub genes exhibited excellent diagnostic performance, with FAM184A showing the best performance. Immune infiltration analysis revealed the relationships between hub gene expression levels and various immune cells. Pan-cancer analysis revealed the expression levels of FAM184A in different tumors, and it showed that high expression of FAM184A in SARC, SKCM, and PAAD is associated with improved prognosis and reduced mortality rates. Finally, RT-qPCR analysis revealed the mRNA expression levels of the hub genes in both OP and CKD. The mRNA expression of all hub genes were downregulated in osteoporosis model mice compared with normal mice, while in CKD mice, the mRNA expression of all hub genes except FAM184A was upregulated.

**Conclusions:**

This study identified four hub genes with significant diagnostic efficacy, suggesting they may act as crucial links between osteoporosis and chronic kidney disease. These genes offer promising targets for the treatment of both diseases. The findings of this study provide valuable insights for future research, which could further elucidate the complex pathogenetic mechanisms connecting chronic kidney disease and osteoporosis.

## 1. Introduction

Osteoporosis (OP) is a systemic skeletal disorder [1], approximately 50% of women and nearly 25% of men aged 50 and older will experience fractures due to OP [2], which is characterized by reduced bone mass and microstructural deterioration of bone tissue, leading to increased bone fragility and susceptibility to fractures [3]. Fractures resulting from osteoporosis usually occur in the spine, hip, or wrist, but other bones may also be affected [4]. Because hospitalization escalates the risk of other medical complications, the mortality rate within 12 months for osteoporotic hip and spine fractures can reach up to 20% [5].

Chronic kidney disease (CKD) is an incurable progressive disease associated with high incidence and mortality rates. It is characterized by structural and functional alterations in the kidneys, leading to kidney failure due to various factors [6]. Although the etiology of CKD remains incompletely understood, hypertension and diabetes mellitus are commonly recognized as its primary reasons [7].

Previous studies have demonstrated a high prevalence of OP among patients with CKD. There are already some mechanisms available to explain this phenomenon. CKD can affect bone density through various substances such as calcium, phosphorus, vitamin D, and parathyroid hormone [8]. However, the impact of these substances on bone density remains controversial. Some studies have shown a negative correlation between vitamin D and bone loss. However, other research indicates that vitamin D levels may not exhibit any association with bone density in CKD patients [9]. CKD can also impact OP through inflammatory reactions; inflammatory factors like IL-1, IL-6, and TNF-α can stimulate bone resorption and inhibit bone formation [10]. In patients with CKD, elevated production of the inflammatory factor IL-6 leads to slowed OP progression [11]. Inflammatory cytokines can further decrease bone metabolism indirectly by suppressing parathyroid hormone [12,13]. Interactions among stress, macromolecular damage, protease inhibition, metabolism, stem cell regeneration, and epigenetics are all implicated in the pathogenesis of OP [14]. Chronic inflammation inevitably leads to bone loss, thereby initiating the onset of OP [15]. Inflammation also stands as a critical risk factor in the progression of CKD [16], exerting adverse effects on CKD through various pathways [17]. An unexpected link exists between CKD and OP [18].

Currently, there are no standardized treatment guidelines for CKD patients with OP. While Kidney Disease: Improving Global Outcomes(KDIGO) guidelines offer some treatment suggestions for CKD patients, they do not provide explicit recommendations on effectively managing OP conditions [19]. The treatment for CKD patients with OP remains challenging and controversial, the treatment of CKD patients with OP typically emphasizes individualized and multidisciplinary approaches [20]. The rapid progress of microarray technology assists in identifying the molecular mechanisms and biomarkers of diseases by analyzing gene or protein expression levels, providing crucial assistance in disease diagnosis and individualized treatment. We aim to use microarray technology to investigate potential common pathways and hub genes between CKD and OP, which could offer prospective therapeutic targets. In recent years, advancements in methodologies such as Weighted Gene Co-expression Network Analysis (WGCNA) and machine learning have significantly progressed, proving to be valuable tools in biomedical informatics for data analysis. WGCNA could identify modules most closely associated with diseases based on variations in gene expression levels [21]. Machine learning uses robust computational algorithms to discern genes strongly linked to diseases [22]. Moreover, CIBERSORT is a versatile computational tool utilized to investigate the composition and functionality of immune cells in various diseases. We use this tool to evaluate the composition of immune cells in CKD and OP [23].

As shown in S1 File, the datasets containing patient and normal samples were downloaded from the GEO platform. Subsequently, we used machine learning algorithms to identify hub genes from common DEGs and assessed their expression levels and diagnostic efficacy by using ROC curves. As our dataset comprised samples derived from peripheral blood mononuclear cells, we downloaded datasets from other tissue sources to conduct immune infiltration analysis, aiming to further elucidate the relationship between hub genes and the distribution of immune cells. The incidence rates of CKD and OP exhibit age correlation, with higher age associated with increased disease prevalence [24]. Consequently, we conducted a pan-cancer analysis to further investigate the role of hub genes in the progression, diagnosis, and treatment of malignant tumors. Lastly, DSigDB database was used to identified candidate drugs related to hub genes.

## 2. Methods

### 2.1. Microarray data

The datasets GSE7158, GSE70528, GSE15072 and GSE56815 were downloaded from the GEO database. Microarray data of GSE15072 generated using the GPL96 [HG-U133A] Affymetrix Human Genome U133A Array, contained 29 samples consisting of 21 CKD samples and 8 control samples. Microarray data of GSE70528 generated using the GPL570 [HG-U133_Plus_2] Affymetrix Human Genome U133 Plus 2.0 Array, contained 19 samples consisting of 11 CKD samples and 8 control samples. Microarray data of GSE56815 generated using GPL96 [HG-U133A] Affymetrix Human Genome U133A Array, contained 40 OP samples and 40 control samples. Microarray data of GSE7158 generated using GPL570 [HG-U133_Plus_2] Affymetrix Human Genome U133 Plus 2.0 Array, contained 12 OP samples and 14 control samples. In this study, samples from patients with OP or CKD were obtained from peripheral blood mononuclear cells. The datasets GSE15072 and GSE56815 were utilized as the training datasets, and the datasets GSE7158 and GSE70528 were utilized as validation datasets.

### 2.2. Identification of differentially expressed genes

The initial raw data was uniformly standardized using the normalizeBetweenArrays function within the “limma” package of R software. The identification of DEGs between OP patients and controls was conducted using the “limma” package of R software (version 4.1.1). The identification of DEGs between CKD patients and controls was performed utilizing the “limma” package of the R software (version 4.1.1). Subsequent analysis was conducted using different packages of the R software. The criteria for identifying DEGs were set as P < 0.05 and |log fold change (FC)|>|0.25|. Volcano plots and heatmaps were used to visually represent the expression outcomes of DEGs. The overlapping genes were obtained by intersecting the DEGs of CKD and OP by using Venn diagram.

### 2.3. Application of WGCNA

WGCNA (Weighted Gene Co-expression Network Analysis) is a method that constructs a gene co-expression network based on gene expression levels. WGCNA categorizes genes into different modules based on their expression levels and can identify modules most closely associated with diseases. Through the exploration of modular gene sets, it aids in understanding gene functionality, identifying biomarkers, and recognizing gene sets associated with specific phenotypes. We constructed the gene co-expression network by using the “WGCNA” package of R software. Samples were clustered to identify and eliminate significant outliers. Subsequently, an automatic network was developed based on co-expression patterns. Following this, we set the soft threshold (β) for calculating adjacency in the co-expression network. Modules with p < 0.05 were selected as matching modules, and the genes within each module were utilized for further analysis.

### 2.4. Analysis of gene ontology, KEGG, and disease ontology for DEGs

GO analysis mainly elucidates gene interactions and coordination in various biological processes. Kyoto Encyclopedia of Genes and Genomes (KEGG) is an integrated database of genomic, chemical, and systemic functional information. KEGG aids in studying the behavior of entire biological systems, rather than focusing on individual genes or proteins. Disease Ontology (DO) analysis is mainly used to identify associations between diseases and genes and understand and help identify potential therapeutic targets. In this study, the functional enrichment analysis of Gene Ontology (GO), Kyoto Encyclopedia of Genes and Genomes (KEGG) and Disease Ontology pathways, was performed using the “clusterProfiler” package of R software. A P-value< 0.05 was considered statistically significant. We conducted DO, GO, and KEGG analyses to investigate the biological functions, signaling pathways, and disease associations potentially related to DEGs or shared DEGs. The common DEGs were further employed in screening the hub genes.

### 2.5. Gene set enrichment analysis

GSEA exhibits exceptional sensitivity in detecting subtle but crucial changes in gene expression. Compared to individual gene differential analysis, it proficiently captures the overall changes within gene sets, aiding in the discovery of overlooked significant signals. GSEA can also assess the enrichment status of gene sets within specific biological functions or pathways by analyzing overall gene expression patterns. Normalized Enrichment Score (NES) and p-values were calculated to identify critical terms for subsequent analyses. The results were computed using the “clusterProfiler” package of R software.

### 2.6. Selection of hub genes

The application of machine learning in the biomedical field is increasingly widespread, enabling effective analysis of biomedical data. In this study, we used four machine learning algorithms (GBM, Random Forest, SVM-RFE, and XGBoost) to predict hub genes among DEGs. The utilized algorithms include GBM, Random Forest, SVM-RFE, and XGBoost. The SVM-RFE algorithm utilizes Support Vector Machine (SVM) for feature selection by iteratively eliminating features with minor impact on classification to enhance model performance. SVM-RFE computations were conducted using the “e1071,” “kernlab,” and “caret” packages of R software. The GBM (Gradient Boosting Machine) algorithm is an ensemble learning method that iteratively trains multiple weak classifiers, progressively enhancing the overall predictive performance of the model by correcting errors made by the previous iterations. The GBM algorithm was conducted using “gbm” package of R software, genes with importance scores >0 were regarded as the hub genes. The XGBoost algorithm is a gradient boosting ensemble learning method that optimizes the predictive performance of decision tree models, incorporating regularization strategies to progressively enhance the model's accuracy and generalization capability. The XGBoost algorithm was conducted using “xgboost” of R software. Random forest model is an ensemble learning model composed of multiple decision trees. It constructs numerous trees by randomly selecting features and samples,and predicts by averaging or voting among these trees to enhance the accuracy and generalization capability of the model. Random forest model was conducted using “randomForest” of R software to perform the computation for predicting hub genes. Using the computational outcomes from four machine learning algorithms, the identification of overlapping genes as hub genes was achieved through a Venn diagram analysis.

### 2.7. The receiver operating characteristic curve analysis

ROC curves are commonly used in evaluating the accuracy of medical diagnostic tests, with True Positive Rate (Sensitivity) on the y-axis and False Positive Rate on the x-axis. The closer the curve approaches the top-left corner, the better the diagnostic performance of the test. We utilized ROC curve analysis to evaluate the diagnostic value of hub genes in CKD and OP. AUC (Area Under the Curve) represents the area under the ROC curve and is used to assess the performance of diagnostic tests. An AUC > 0.5 indicates a certain level of accuracy and reliability for the diagnostic test, as the AUC approaches 1, it signifies improved performance of the diagnostic test, indicating its higher accuracy in distinguishing whether patients have a particular disease or condition. The plotting of ROC curves was implemented using the ‘pROC’ package of R software.

### 2.8. Immune infiltration analysis

Immune cells play an important role in the progression of diseases. Examining the components of immune cells can help unveil the underlying pathogenic mechanisms of the diseases. CIBERSORT, a computational tool used to analyze the composition of immune cells within tissues, estimates the relative abundance of distinct immune cell subtypes in samples based on gene expression data through machine learning algorithms. In this study, we used CIBERSORT version 0.1.0,with signature matrix was the built-in `LM22.txt` in CIBERSORT, which contains the gene expression signatures of 22 types of human immune cells.Quantile normalization (QN) parameter was set to `F`; All other parameters were set to the default values of the CIBERSORT function to ensure the standardization and reproducibility of the analysis process. The visualization of immune infiltration results is presented through boxplots, line graphs, and heatmaps

### 2.9. Pan-cancer analysis

Pan-cancer analysis is a comprehensive oncogenomic approach involving high-throughput sequencing and analysis of tumor samples to elucidate the hereditary characteristics, mutation patterns, and genomic variations associated with cancers. Encompassing various cancer types, pan-cancer analysis aims to identify common oncogenic features and alterations, facilitating the development of more precise individualized therapeutic strategies for patients. After excluding cancer data sets with fewer than 5 samples, we extracted gene expression data for each sample from a standardized pan-cancer dataset comprising 33 cancer types. Gene expression values were log2-transformed using the formula log2(x + 1), and differential analysis was conducted utilizing non-matched Kruskal-Wallis tests. The ‘coxph’ function from the R package ‘survival’ was used to establish Cox proportional hazards regression models, examining the correlation between gene expression in each tumor and prognosis. Log-rank tests were used as statistical tests to ascertain prognostic significance.

### 2.10. Candidate drugs

The Drug Signatures Database (DSigDB) comprised information on thousands of drugs, including their gene expression patterns, interactions with targets, and effects on cells. These data may contribute to the exploration of novel therapeutic strategies. We use the Enrichr platform to get access to the Drug Signatures Database.

### 2.11. Animal experiment

A total of 20 C57BL/6J female mice were purchased from Leagene Biotechnology Co., Ltd. (Beijing, China). The mice were housed in a facility with a 12-hour light-dark cycle, maintained at a temperature of 23 ± 1°C, and appropriate humidity levels. Throughout the study, the animals had ad libitum access to food and water. All animal experiments were conducted in accordance with the relevant guidelines and approved by the Animal Ethics Committee of Changzheng Hospital, the Second Affiliated Hospital of Naval Medical University. All methods were reported in compliance with the ARRIVE guidelines. 10 female C57BL/6J mice were randomly assigned to two groups: the Control group and the CKD group. Mice in the CKD group were administered 2.5 mg/kg cisplatin via intraperitoneal injection for five consecutive days; on day 21, cisplatin was re-administered for an additional five days. On day 41, the mice were anesthetized with 50 mg/kg sodium pentobarbital, blood samples were collected, and euthanasia was performed via cervical dislocation. The Control group received saline injections and underwent the same procedures as the experimental group, with no other differences. The remaining ten female C57BL/6J mice were randomly assigned to the control group and the osteoporosis group. Mice in the OP group were anesthetized by intraperitoneal injection of 1% sodium pentobarbital (50 mg/kg) and placed in a supine position on the operating table. After routine disinfection of the abdominal surgical area, a 1.5 cm midline incision was made, and the abdominal tissues were dissected layer by layer to fully expose the bilateral ovaries and surrounding adipose tissue. The ovaries were carefully isolated, ligated at the ovarian pedicles, and completely excised. The muscle and skin layers were then sutured sequentially. Mice in the control group underwent the same surgical procedures without removal of the ovaries (sham operation). After surgery, all mice were maintained under standard conditions for 12 weeks. Subsequently, the mice were anesthetized with sodium pentobarbital (50 mg/kg), blood samples were collected, and euthanasia was performed by cervical dislocation.

### 2.12. Real-time quantitative PCR

Total RNA was extracted from Peripheral Blood Mononuclear Cells using TRIzol (Thermo Fisher Scientific). RNA concentration was measured using an ultra-micro spectrophotometer. The RNA was then reverse transcribed into complementary DNA (cDNA) using a reverse transcription kit. Subsequently, RT-qPCR was performed using the SYBR Green Pro Taq HS Premixed qPCR Kit (Accurate Biology). Finally, the relative quantification method was applied to calculate the results with Gapdh as the reference gene. Primer sequences are listed in Table 1. The primers were synthesized by Shanghai Sangon Biotechnology Co.,Ltd.

**Table 1:** RT-qPCRprimers’ list.

Gene	Forword	Reverse
FAM184A	GGAGACAAGCTGACTTCAGCCA	GCTCACTCTGAAGCCTTTCCTG
NFKBIA	GCCAGGAATTGCTGAGGCACTT	GTCTGCGTCAAGACTGCTACAC
RP2	CAGTGATGAATCATGTCTTGTGGT	GTCTGCACTAGGGAGAAGCCTT
HIRA	TCACAGAACGGTCCAAAGCCAC	CTCCTTGACGAGGTTCTGCTCT
GAPDH	CATCACTGCCACCCAGAAGACTG	ATGCCAGTGAGCTTCCCGTTCAG

### 2.13. Statistical analysis

All data were comprehensively analyzed using GraphPad Prism 9.0 software. The t-test was used to analyze the experimental data, and a P value of < 0.05 was considered statistically significant

## 3. Results

### 3.1. Identification of DEGs

**Fig 1:**
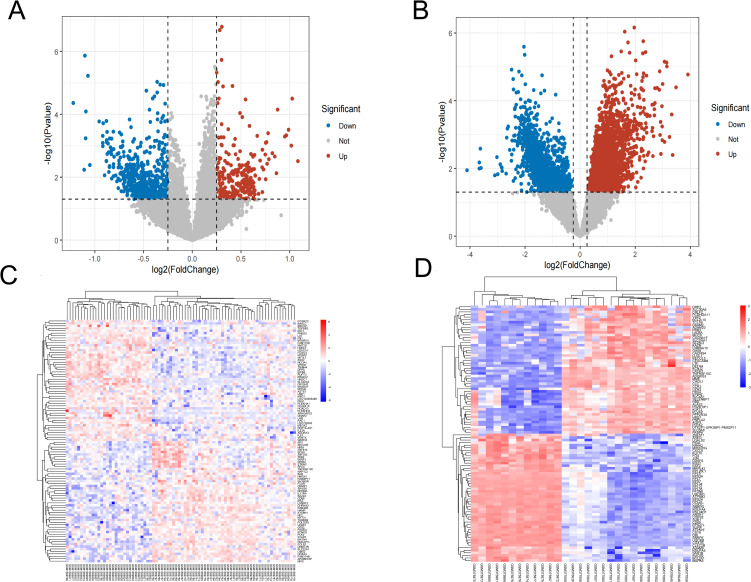
Screening of DEGs. volcano plots(A,B) and Heatmap(C,D) depicting the DEGs of the OP and CKD datasets. **(A)** DEGs were identified between OP patients and normal individuals, the upregulated DEGs were highlighted in red and the down-regulated DEGs were highlighted in blue. **(B)** DEGs were identified between CKD patients and normal individuals, the upregulated DEGs were highlighted in red and the downregulated DEGs were highlighted in blue. **(C)** The heatmap showed the top 50 upregulated DEGs and downregulated DEGs between OP patients and normal individuals, color intensity reflects the levels of gene expression. **(D)** The heatmap showed the top 50 upregulated DEGs and downregulated DEGs between CKD patients and normal individuals, color intensity reflects the levels of gene expression.

Using the “limma” package of R software, a total of 795 DEGs were identified in the OP dataset (GSE56815). Among the 795 DEGs, there were 260 upregulated genes and 535 downregulated genes. In the CKD dataset (GSE15072), a total of 4336 genes were screened, with 2190 genes exhibiting upregulation and 2146 genes showing downregulation. The DEGs from the OP dataset and CKD dataset were visually represented using the volcano plots (Fig 1A, B) and the heatmaps (Fig 1C, D) to illustrate gene expression levels.

### 3.2. Construction and analysis of WGCNA

**Fig 2:**
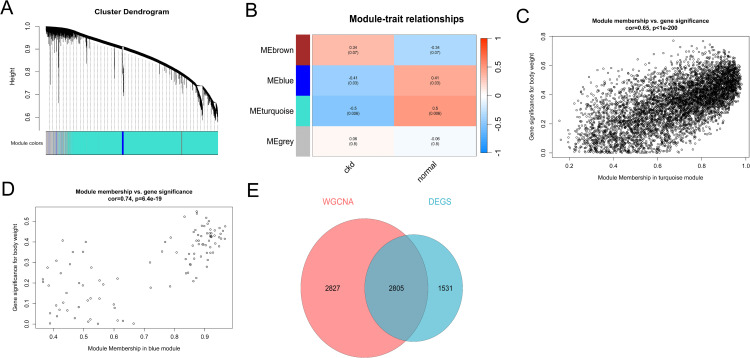
Weighted Gene Co-expression Network Analysis. **(A)** The modules with different colors were presented in the clustering tree. **(B)** Heatmap showed the module-traits relationship. **(C)** MM versus GS scatter plot of turquoise module **(D)** MM versus GS scatter plot of blue module. **(E)** Venn diagram between module genes and DEGs resulted in 2805 common genes in CKD dataset.

According to scale independence, we set the value of the soft threshold power to 8. A total of 29 samples (21 CKD patients and 8 healthy controls) were included in the WGCNA analysis. A hierarchical clustering dendrogram was constructed, identifying four modules exhibiting distinct characteristics (Fig 2A). Subsequently, we explored the relationship between modules and clinical groups (normal controls and CKD patients) to identify key module genes potentially associated with CKD. The turquoise module (r = −0.5, p = 0.006) and blue module (r = −0.41, p = 0.03) demonstrated significant negative correlations with CKD (Fig 2B). In the MM versus GS scatter plot, a great correlation with CKD was observed. A total of 5632 genes from these two modules were identified (Fig 2C, D). We subsequently used a Venn diagram to select the overlapping genes between module genes and DEGs in CKD dataset for further analysis (Fig 2E).

### 3.3. Functional enrichment analysis of DEGs

To evaluate the reliability of the GSE56815 dataset regarding the pathogenesis of OP, we performed functional enrichment analysis on the DEGs of the GSE56815 dataset for validation.

**Fig 3:**
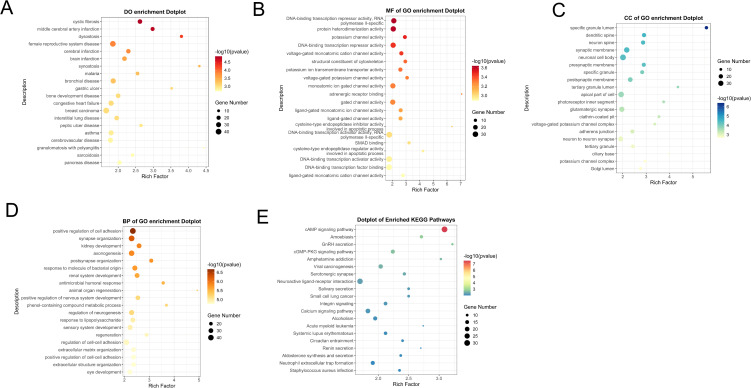
Functional enrichment analysis of DEGs in OP dataset. **（A）**DO enrichment analysis of DEGs **(B-D)** GO enrichment analysis of DEGs in the BP, CC and MF categories. **(B)** KEGG enrichment analysis of DEGs.

A total of 795 DEGs were selected for functional enrichment analysis. The DO analysis results revealed potential associated diseases, including “cystic fibrosis” “middle cerebral artery infarction” “dysostosisfemale reproductive system disease” “cerebral infarction” “brain infarction” “synostosis” “malaria” “bronchial disease” and “gastric ulcer” (Fig 3A). The GO analysis results showed that the top five molecular functions were “DNA-binding transcription repressor activity, RNA polymerase II-specific,” “protein heterodimerization activity,” “potassium channel activity,” “DNA-binding transcription repressor activity” and “voltage-gated monoatomic cation channel activity”(Fig 3B). The GO analysis results showed that the top five cell components were “specific granule lumen,” “dendritic spine,” “neuron spine,” “synaptic membrane” and “neuronal cell body”(Fig 3C). The GO analysis results showed that the top five biological processes were “positive regulation of cell adhesion,” “synapse organization,” “kidney development” and “axonogenesis,” “postsynapse organization”(Fig 3D). The KEGG results demonstrated significant enrichment of genes within specific pathways including “cAMP signaling pathway,” “Amoebiasis,” “GnRH secretion,” “cGMP-PKG signaling pathway,” and “Amphetamine addiction” (Fig 3E). The functional enrichment analysis revealed that the 795 DEGs in the OP dataset are associated with inflammatory responses.

**Fig 4:**
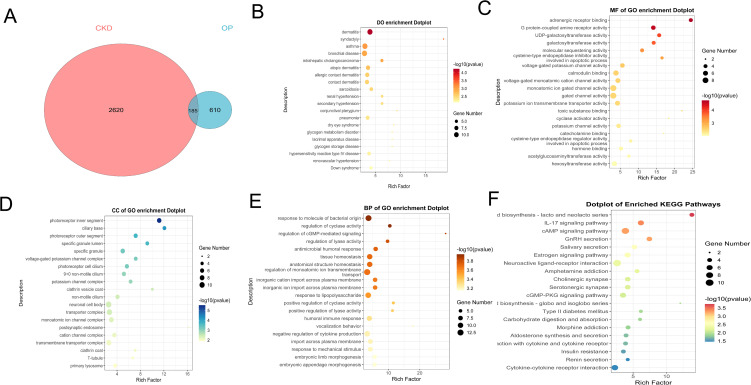
Functional enrichment analysis of common DEGs. **（A）**Venn diagram showed 185 common DEGs. **(B)** DO enrichment analysis of common DEGs **(C-E)** GO enrichment analysis of common DEGs in the BP，CC and MF categories. **(B)** KEGG enrichment analysis of common DEGs.

Using a Venn diagram (Fig 4A), we identified the common DEGs for further research. A total of 185 common DEGs were identified and selected. We conducted GO, KEGG, and DO analyses on these overlapping genes to explore their significance and potential roles. The DO analysis results revealed potential associated diseases, including “dermatitis” “syndactyly” “asthma” “bronchial disease” “intrahepatic cholangiocarcinoma” “atopic dermatitis” “allergic contact dermatitis” “contact dermatitis” “sarcoidosis” and “renal hypertension” (Fig 4B). The GO analysis results showed that the top five molecular functions were “adrenergic receptor binding,” “G protein-coupled amine receptor activity,” “UDP-galactosyltransferase activity,” “galactosyltransferase activity” and “molecular sequestering activity” (Fig 4C). The GO analysis results showed that the top five cell components were “photoreceptor inner segment,” “ciliary base,” “photoreceptor outer segment,” “specific granule lumen” and “specific granule” (Fig 4D). The GO analysis results showed that the top five biological processes were “response to molecule of bacterial origin,” “regulation of cyclase activity,” “regulation of cGMP-mediated signaling,” “regulation of lyase activity” and “antimicrobial humoral response” (Fig 4E). The KEGG results demonstrated significant enrichment of genes within specific pathways including “Glycosphingolipid biosynthesis – lacto and neolacto series,” “IL-17 signaling pathway,” “GnRH secretion,” “cAMP signaling pathway” and “Salivary secretion” (Fig 4F).

### 3.4. Gene set enrichment analysis

**Fig 5:**
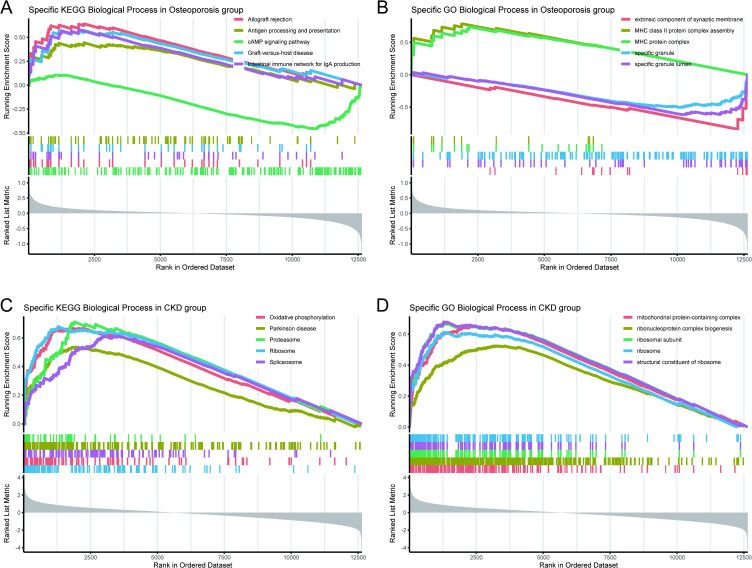
GSEA of CKD and OP group. (A) specific GO biological process by GSEA in OP group (B) specific KEGG biological process by GSEA in OP group (C) specific GO biological process by GSEA in CKD group (D) specific KEGG biological process by GSEA in CKD group.

The results of GSEA of KEGG biological process in OP group showed that the top five pathways were “Allograft rejection” “Antigen processing and presentation” “cAMP signaling pathway” “Graft-versus-host disease” and “intestinal immune network for IgA production” (Fig 5A). The top five GO biological processes in osteoporosis group by GSEA were “calcium ion homeostasis” “extrinsic component of synaptic membrane” “MHC protein complex” “specific granule” and “specific granule lumen” (Fig 5B). The KEGG function enrichment analysis indicated that “chemical carcinogenesis-reactive oxygen species” “oxidative phosphorylation” “parkinson disease” “ribosome” and “Spliceosome” were related to CKD group (Fig 5C). The top five GO biological processes in CKD group by GSEA were “mitochondrial protein-containing complex” “ribonucleoprotein complex biogenesis” “ribosomal subunit” “ribosome” and “structural constituent of ribosome” (Fig 5D).

### 3.5. Identification of hub genes by machine learning

**Fig 6:**
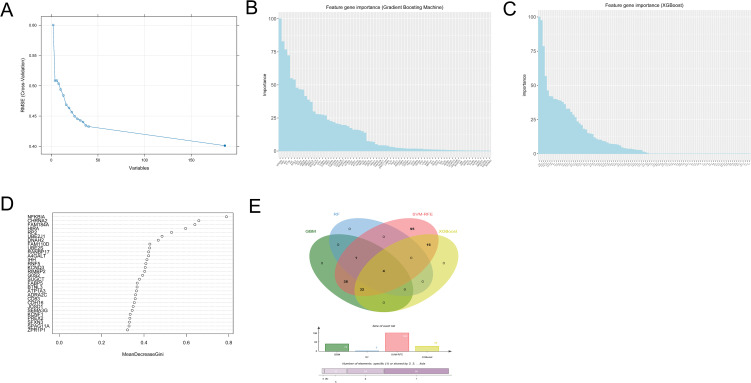
Machine learning to select hub genes. **(A)** Screening of candidate diagnostic genes using SVM-RFE algorithm. **(B)** Screening of candidate diagnostic genes using GBM algorithm, and the bar chart depicted the ranked importance of genes. **(C)** Screening of candidate diagnostic genes using XGBoost algorithm, and the bar chart depicted the ranked importance of genes. **(D)** The relationship between the number of classification trees and error rate in random forests. **(E)** Selecting the overlapping genes among the four machine learning algorithms using Venn diagram.

The identification of hub genes was conducted using four machine learning algorithms. 186 genes were identified by using SVM-RFE algorithm (Fig 6A). GBM algorithm identified 75 genes by feature gene importance (Fig 6B). Fig 6C showed that a total of 52 genes were identified by using XGBoost algorithm. Utilizing MeanDecreaseGini > 0.5 as the criterion, the random forest algorithm identified 5 genes (Fig 6D). We used a Venn diagram to visually present the overlapping genes among the identification of the four machine learning algorithms (Fig 6E). A total of four hub genes were identified for further study (“NFKBIA,” “RP2,” “HIRA,” “FAM184A”).

### 3.6. Validation of the diagnostic values of hub genes

**Fig 7:**
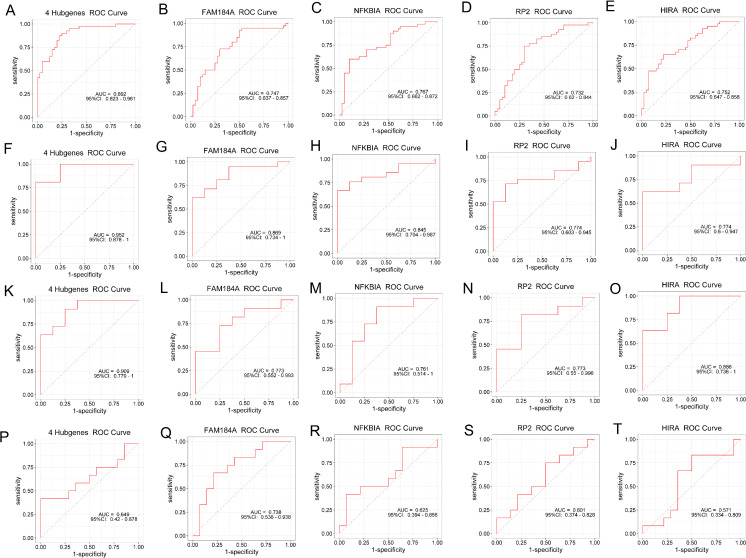
Validation of hub genes in diagnosing diseases. **(A-E)** OP training dataset **(F-J)** CKD training dataset **(K-O)** CKD validation dataset **(P-T)** OP validation dataset.

**Fig 8:**
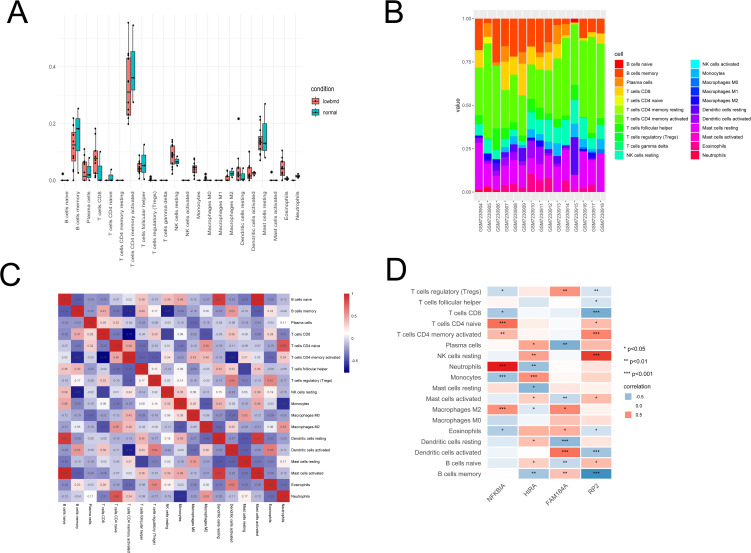
Immune infiltration analysis. **(A)** The components of 22 immune cells in OP patient samples were visualized using the box plot **(B)** The components of 22 immune cells in OP patient samples were visualized using the bar plot **(C)** Correlation among the components of the 18 immune cell types in OP patient samples. **(D)** Correlation between 18 immune cell expressions and hub Genes.

The ROC curves were plotted to assess the diagnostic efficacy of the four hub genes (Fig 7). In the OP training datasets (GSE56815), the four hub genes had significant diagnostic performance (AUC:0.892) (Fig 7A). The individual diagnostic performance of the four hub genes also demonstrated excellent efficacy: FAM184A (AUC:0.747), NFKBIA (AUC:0.767), RP2 (AUC:0.732), HIRA (AUC:0.752) (Fig 7B-8E). Within the CKD training datasets (GSE15072), the four hub genes also exhibited great diagnostic efficacy (AUC: 0.952) (Fig 7F). The independent diagnostic capabilities of the four hub genes also showed great performance: FAM184A (AUC:0.869), NFKBIA (AUC:0.845), RP2 (AUC:0.774), HIRA (AUC:0.774) (Fig 7G-7J). We subsequently downloaded GSE70528 and GSE7158 as the validation datasets to further analysis. The four hub genes had great diagnostic abilities in OP validation dataset (AUC:0.909) (Fig 7K). Based on the AUC values, each of the four hub genes showed significant capability for diagnosing CKD: FAM184A (AUC:0.773), NFKBIA (AUC:0.761), RP2 (AUC:0.773), HIRA (AUC:0.886) (Fig 7L-7O). In the validation datasets, the diagnostic performance of the four hub genes for OP was slightly less effective compared to their performance in diagnosing CKD(AUC:0.649) (Fig 7P). The AUC value of FAM184A for diagnosing OP was 0.738 while other genes showed no significant diagnostic ability in OP (Fig 7Q-7T)

### 3.7. Immune infiltration analysis and the correlation between hub genes and immune cells

The immune microenvironment comprises a combination of various immune cells, cytokines, and signaling molecules, playing a crucial role in disease progression and treatment, impacting disease advancement and therapeutic responses. Therefore, conducting immune infiltration analysis can better help our understanding of disease diagnosis, progression, prognosis, and treatment. We conducted an assessment of the immune profiles in both normal and OP patient samples to discern variations in immune levels between the two states (Fig 8A-8D). In contrast to OP patient samples, the normal samples exhibited significant differences in the expression levels of B cell memory, T cells CD8, T cells CD4 memory activated, NK cells resting, Monocytes, Macrophages M2, and Eosinophils (Fig 8A,8B). Subsequently, we conducted analysis to determine the correlation of the immune cell components in OP patient samples. As shown in Fig 8C, there was a significant positive correlation observed between native B cells and resting dendritic cells resting with activated mast cells. B cells also had a significant positive correlation with dendritic cells resting. In addition, there was a significant negative correlation observed between T cells follicular helper and Mast cells resting. The relationship between the four hub genes and immune cell expression was depicted in Fig 8D. Neutrophils, T cells CD4 naive and Macrophages M2 were notable positively correlated with NFKBlA. Conversely, Monocytes were significantly negatively correlated with NFKBlA. Interestingly, Monocytes were positively correlated with HIRA. In addition, Dendritic cells activated showed remarkable positive correlation with FAM184A, but Dendritic cells resting showed remarkable negative correlation with FAM184A. RP2 presented a significant positive correlation with resting NK cells and activated T cells CD4 memory. Conversely, there was a significant negative correlation observed between RP2 and memory B cells, activated dendritic cells, and CD8 T cells.

### 3.8. Pan-cancer FAM184A expression

**Fig 9:**
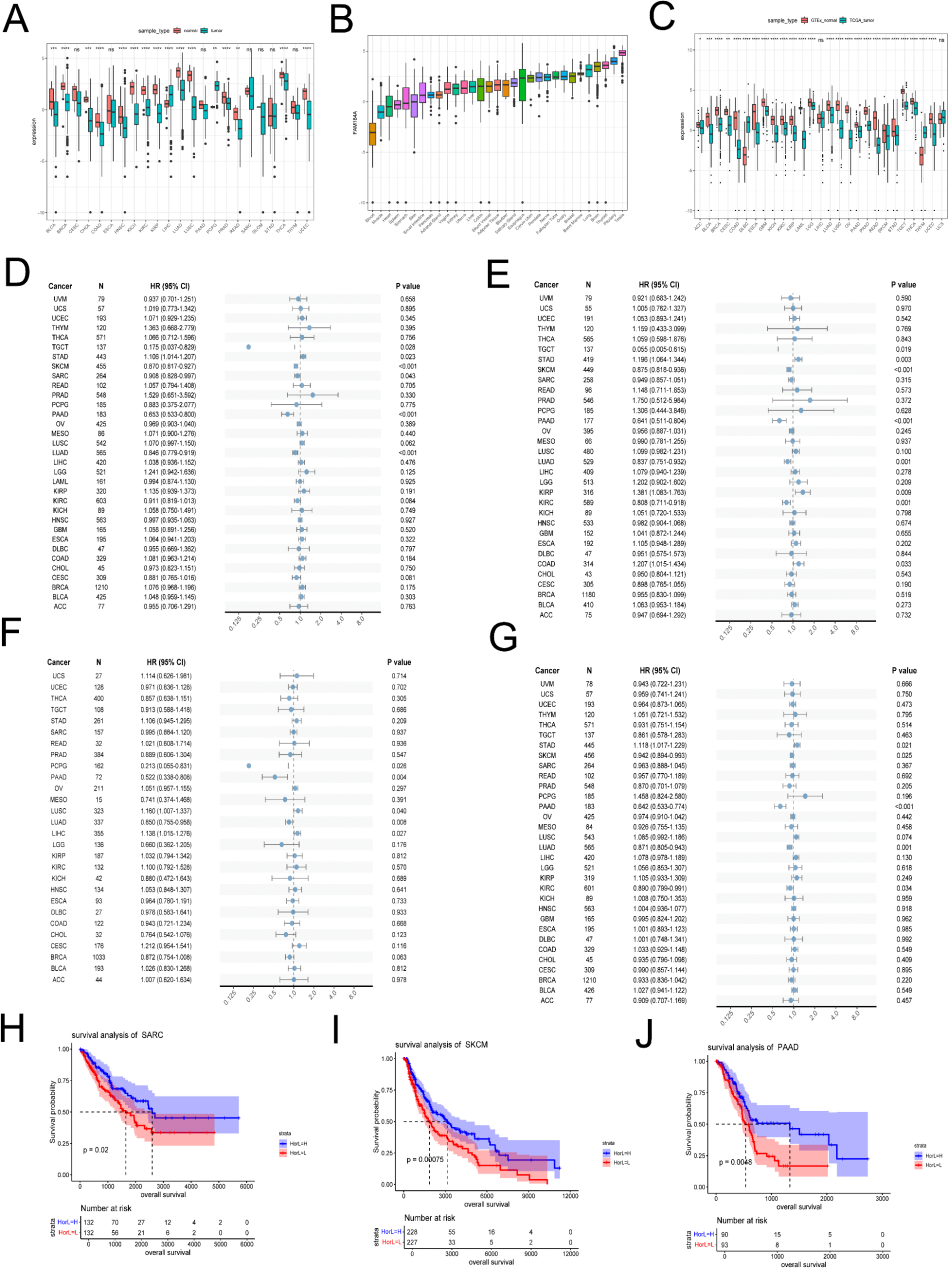
The expression of FAM184A in Pan-cancer. **(A)** Pan-cancer expression levels of FAM184A in the TCGA dataset. **(B)** The normal samples expression levels of FAM184A in the GTEx datasets **(C)** Pan-cancer expression levels of FAM184A in the TCGA and GTEx datasets. **(D)** Forest plots of FAM184A expression and OS **(E)** Forest plots of FAM184A expression and DSS **(F)** Forest plots of FAM184A expression and DFI **(G)** Forest plots of FAM184A expression and PFI. **(H)** Higher expression levels of FAM184A were associated with superior overall survival in SARC. **(I)** Higher expression levels of FAM184A were associated with superior overall survival in SKCM. **(J)** Higher expression levels of FAM184A were associated with superior overall survival in PAAD. *p < 0.05, **p < 0.01, ***p < 0.001, ****p < 0.0001, ns- no significance.

We firstly computed the expression differences between normal and tumor samples in each tumor. In 23 TCGA tumor types with controls, two tumor exhibited significant upregulation of FAM184A, including Liver Hepatocellular Carcinoma (LIHC) and PCPG Pheochromocytoma and Paraganglioma(PCPG）,while 14 tumors exhibited significant downregulation of FAM184A,including Bladder Urothelial Carcinoma(BLCA), Breast Invasive Carcinoma(BRCA), Cholangiocarcinoma (CHOL),Colon Adenocarcinoma(COAD), Head and Neck Squamous cell carcinoma(HNSC), Kidney Chromophobe (KICH), Kidney renal clear cell carcinoma (KIRC), Kidney renal papillary cell carcinoma (KIRP), Lung adenocarcinoma (LUAD), Lung squamous cell carcinoma (LUSC), Rectum adenocarcinoma (READ), Prostate adenocarcinoma (PRAD), Thyroid carcinoma (THCA) and Uterine Corpus Endometrial Carcinoma (UCEC) (Fig 9A).

We downloaded the normal samples from GTEx datasets and conducted difference analysis to validate the results with tumor samples. The Fig 9B showed the FAM184A expression in normal samples and the Fig 9C showed the results of difference analysis between the normal samples and tumor samples. FAM184A exhibited significantly high expression in two types of tumors: DLBC and THYM. While FAM184A exhibited significantly low expression in 22 types of tumors: Adrenocortical carcinoma (ACC), Bladder Urothelial Carcinoma (BLCA), Cervical squamous cell carcinoma and endocervical adenocarcinoma (CESC), Colon adenocarcinoma (COAD), Esophageal carcinoma (ESCA), Glioblastoma Multiforme (GBM), Kidney Chromophobe (KICH), Kidney renal clear cell carcinoma (KIRC), Kidney renal papillary cell carcinoma (KIRP), Acute Myeloid Leukemia (LAML), Brain Lower Grade Glioma (LGG), Lung adenocarcinoma (LUAD), Lung squamous cell carcinoma (LUSC), Ovarian serous cystadenocarcinoma (OV), Pancreatic adenocarcinoma (PAAD), Prostate adenocarcinoma (PRAD), Pediatric Early-Stage Disseminated High-grade Gliomas (PEAD), Skin Cutaneous Melanoma (SKCM), Stomach adenocarcinoma (STAD), Testicular Germ Cell Tumors (TGCT), Thyroid carcinoma (THCA), Uterine Corpus Endometrial Carcinoma (UCEC), According to the results, we found FAM184A had a high level of expression in tumors associated with immune system. However, in other types of tumor tissues, FAM184A showed lower expression levels compared to normal samples. Interestingly, the results showed FAM184A was downregulated in tumors associated with females (OV, CESC and BLCA) in the dataset. Tumors associated with the digestive system and respiratory system also exhibited a low expression level of FAM184A (COAD, ESCA, PAAD, STAD, LUAD and LUSC).

To further investigate the role of low FAM184A expression in cancer risk among patients, we conducted research on the association between FAM184A and the prognosis of pan-cancer. Among these pan-cancer types, we calculated survival time to validate the prognosis in pan-cancer, including overall survival (OS), disease-specific survival (DSS), disease-free interval (DFI), and progression-free interval (PFI). TGCT, SKCM, SARC, PAAD and LUAD had lower OS time and worse prognosis with FAM184A low expression (Fig 9D). However, STAD was related to high death risk and worse prognosis with FAM184A high expression (Fig 9D). TGCT, SKCM, PAAD, LUAD and KIRC had higher DSS time with low FAM184A expression (Fig 9E), while STAD, LCG and COAD had lower DSS time with high FAM184A expression (Fig 9E). The results of DFI and PFI were shown in Fig 9F, 9G. Subsequently, we draw K-M curve of tumors to further demonstrate the relationship between tumors and FAM184A low expression. In addition, SARC, SKCM and PAAD had better prognosis and lower death risk with FAM184A high expression (Fig 9H-G).

### 3.9. identification of candidate drugs

We used the DSigDB database by the Enrichr platform to discover the small molecule drugs that regulated the expression of hub genes. The identification of potential small molecule candidate drugs was based on their P-values. the top 10 potential small molecule candidate drugs were showed in Table 2.

**Table 2:** List of the top 10 suggested drugs for patients with both osteoporosis and chronic kidney disease.

Name	P-value	Odds Ratio	Combined score
DIGOXIGENIN	0.0008751	81.63	574.76
THIOPENTAL	0.002198	666.2	4077.19
BERBAMINE	0.002198	666.2	4077.19
OSTHOLE	0.002398	605.61	3653.7
IMD-0354	0.002598	555.11	3304.67
AMIFOSTINE	0.002598	555.11	3304.67
1-BROMOPROPANE	0.002598	555.11	3304.67
DENSPM	0.002598	555.11	3304.67
DIOSGENIN	0.002997	475.76	2764.28
MELAMINE	0.002997	475.76	2764.28

### 3.10. Validation of hub genes using RT-qPCR

**Fig 10:**
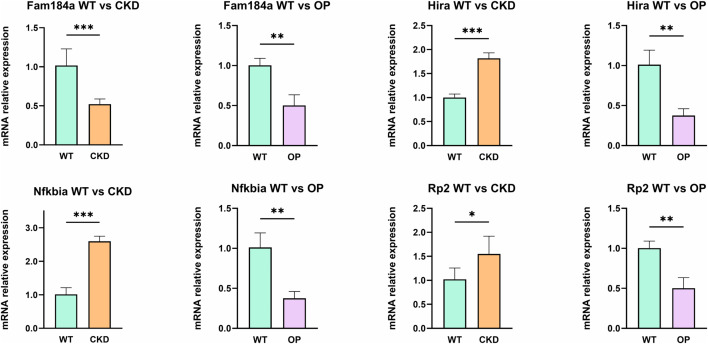
The mRNA expression levels of hub genes, *p < 0.05, **p < 0.01, ***p < 0.001.

We further evaluated the differences in the mRNA expression levels of hub genes between the control group, OP group, and CKD group (Fig 10). RT-qPCR results showed that, compared with normal mice, the mRNA expression levels of all hub genes were downregulated in the osteoporosis model mice. In contrast, in the CKD mice, the mRNA expression of all hub genes, except for FAM184A, was upregulated. This trend is consistent with the results of the bioinformatics analysis.

## 4. Discussion

OP and CKD often coexist in the elderly. CKD often influences bone health by affecting mineral metabolism, leading to an increased risk of fractures [25]. Some studies have already proposed hypotheses about the common pathogenic mechanisms between osteoporosis and chronic kidney disease. Previous research has suggested that bone metabolism may have a significant impact on osteoporosis. Chronic Kidney Disease can disrupt bone metabolism balance by affecting the absorption and secretion of substances such as calcium, phosphate, vitamin D, and PTH [26]. The latest research found that osteocyte dysfunction is linked to various bone abnormalities observed in patients with chronic kidney disease. Dysregulation in osteocyte morphology and signaling has been observed at the earliest stages of CKD, potentially accelerating the progression of kidney disease. This dysfunction may further lead to the advancement of bone diseases such as osteoporosis [27]. These studies have enhanced our understanding of the common pathogenic mechanisms between osteoporosis and chronic kidney disease. However, previous research has primarily focused on the cellular and molecular mechanisms, rather than the gene expression level, to explore the common pathogenesis of osteoporosis and chronic kidney disease. As many medications may exacerbate renal damage in chronic kidney disease, there is still a lack of drugs with high-level evidence of efficacy for patients with osteoporosis coexisting with chronic kidney disease. Bisphosphonates are widely used in osteoporosis therapy, but they can significantly worsen renal function decline [28–30]. Therefore, investigating the pathogenesis of chronic kidney disease and osteoporosis in the genetic level may facilitate the development of personalized and precision medications.

Computer science plays a crucial role in the field of bioinformatics, it can effectively process numerous data of bioinformatics and provide help in the analysis of gene and protein sequences. Utilizing various algorithms and computational models, it enables the identification of disease-related genes. Therefore, we firstly downloaded data from public databases and used machine learning algorithms to identify hub genes, evaluating their diagnostic efficacy in osteoporosis and chronic kidney disease. In this study, we identified four hub genes: NFKBIA, RP2, HIRA and FAM184A. All of them exhibit a certain degree of diagnostic efficacy, with FAM184A demonstrating the highest diagnostic value.

NFKBIA is a member of the NF-κB family, it can regulate various signaling pathways, including Toll-like receptor signaling pathway, adipocytokine pathway, neurotrophin signaling pathway, NOD-like receptor signaling, and B cell receptor signaling pathway [31]. Mutations in NFKBIA can lead to impaired degradation of IkBa, resulting in immune deficiency and impaired dependent inflammatory responses [32]. NFKBIA also is a tumor suppressor, and its deficiency may also be associated with tumor progression and recurrence [33]. The previous study has shown that the downregulation of NFKBIA can attenuate the nuclear factor kappa B (NF-κB) signaling pathway, leading to osteoclastogenesis, which may potentially impact osteoporosis [34]. Moreover, NFKBIA can cause renal dysfunction by influencing chronic inflammatory responses, leading to chronic kidney disease [35]. The RP2 gene contains five exons and produces a protein consisting of 350 amino acids, exhibiting a broad expression pattern. In previous studies, the majority of studies have focused on the relationship between the RP2 gene and X-linked retinitis pigmentosa (XLRP), ultimately confirming that XLRP is caused by RP2 gene mutations. The protein encoded by RP2 has played a crucial role in the photoreceptor cells of the retina. These cells detect light and convert it into neural signals. Mutations in the RP2 gene can lead to impaired function of these cells, ultimately resulting in vision loss [36,37]. In photoreceptor cells, cilia primarily facilitate the transport and localization of proteins within these cells. The RP2 protein can affect the normal function of cilia by acting as the GTPase-activating protein (GAP) for Arl3 [38,39]. However, dysfunction of cilia is also a common cause of chronic kidney disease. Recent studies have discovered that RP2 is located in the primary cilia of renal epithelial cells and can regulate ciliary transport by interacting with the gene product of polycystic kidney disease 2 (PKD2), polycystin-2 [40]. Although there is no direct evidence that RP2 can affect the development of skeletal-related diseases, some research have demonstrated the important role of cilia in regulating osteoblast function and bone defect healing [41,42]. Moreover, the latest studies have shown that osteoblasts from patients with PKD mutations exhibit abnormal cilia, further suggesting that the RP2 gene may influence both kidney function and bone metabolism by regulating cilia [43]. HIRA is a histone chaperone that mainly facilitates the folding and assembly of histones. Some research showed that the HIRA protein was associated with tissue fibrosis, cellular senescence, and tumor development [44–46]. Renal fibrosis is an extremely complex and dynamic process that involves nearly all types of cells in the kidneys and often occurs in chronic kidney disease [47].

The recent research has demonstrated that histone H3.3 and HIRA co-immunoprecipitation are promoters related to renal fibrosis. TGF-β1 can upregulate the expression of histone H3.3 and HIRA through the smad3-dependent pathway, and inhibiting HIRA can reduce fibrosis in renal epithelial cells. The expression of histone H3.3 and HIRA is not only associated with the degree of renal fibrosis but also correlates with eGFR. Targeting HIRA inhibition may be a potential therapeutic strategy for renal fibrosis in chronic kidney disease in the future [48]. Although HIRA plays a key role in cell cycle regulation, DNA damage repair, and gene expression modulation, the direct link between HIRA and osteoporosis is not very clear. Further research should focus on elucidating the relationship between HIRA and osteoporosis. The functions and biological roles of FAM184A are still under exploration, and there are few studies that can associate it with specific diseases. However, some research indicates that it may be linked to some cancers, suggesting its potential research value [49,50]. In this study, FAM184A demonstrated the best performance in the diagnosis of osteoporosis and chronic kidney disease. Therefore, we selected FAM184A for pan-cancer analysis in the subsequent research.

Previous research has predominantly attributed the onset of osteoporosis to estrogen deficiency, aging, or medication side effects. However, more evidence suggests that immune cells may contribute to osteoporosis by secreting inflammatory cytokines and related ligands, which affect bone formation and resorption. Although advancements in the field of bone immunology have enhanced our understanding of the relationship between the skeletal and immune systems, further research still need to focus on identifying immune cells with osteoporosis-inducing potential. This will provide novel therapeutic approaches for osteoporosis [51–54]. Therefore, we conducted immune infiltration analysis in OP patients and control groups to find possible immune cells related to hub genes. In OP patients, NFKBIA showed a significant positive relationship with neutrophils and M2 macrophages. Conversely, a significant negative correlation was observed between NFKBIA expression and monocytes. These innate immune cells have been proved to have an important impact on osteoporosis. Neutrophils can promote inflammatory bone loss by producing chemokines that recruit pro-inflammatory cells. Some studies have demonstrated that activated neutrophils can facilitate the formation and maturation of osteoclasts by secreting RANKL [55]. Monocytes are precursors to macrophages, both of them are innate immune cells derived from the bone marrow lineage. Peripheral blood monocytes (PBMs) can express ANXA2 protein, which facilitates the migration of these monocytes to sites of bone resorption, leading to an enhancement in bone resorption. However, non-activated monocytes can promote bone formation by facilitating the recruitment of mesenchymal stem cells (MSCs) and enhancing their osteogenic potential [53]. M2 macrophages typically play an important role in anti-inflammatory and immunomodulatory functions by secreting anti-inflammatory factors. These cells promote bone mineralization by stimulating the differentiation of mesenchymal stem cells (MSCs) and osteoblast precursors into mature osteoblasts [56]. In this study, the expression of FAM184A has been found to have a strong correlation with dendritic cells in OP patients. Current research indicates that dendritic cells can promote osteoclast formation through the production of pro-inflammatory cytokines. Additionally, in the presence of the receptor activator of nuclear factor kappa-B ligand (RANKL), dendritic cells can also transdifferentiate into osteoclasts, contributing to bone resorption. Newly formed osteoclasts have the capability to induce the chemotaxis of additional dendritic cells, thus creating a self-reinforcing cycle of osteoclast and dendritic cell interaction. This cycle plays a significant role in the process of bone resorption [57]. Comparing with the other hub genes, the expression of RP2 is mainly associated with specific immune cells including activated CD4 memory T cells, naïve CD4 T cells and memory B cells in this study. Based on cytokine expression profiles, CD4 + T cells can be differentiated into various subsets, including Th1, Th2, and Th17 cells. Previously, Th1 cells were often considered to be involved in bone loss. However, recent studies indicate that Th1 cells may play a dual role in osteoclastogenesis [58,59]. Current evidence indicates that Th2 cells may exhibit anti-inflammatory effects through activating B cells. Research has shown that active Th2 cells can maintain the activity of osteoblasts by enhancing the production of parathyroid hormone (PTH), but studies exploring the relationship between Th2 cells and osteoclasts are still limited [60]. The prevailing view is that Th17 cells primarily promote osteoclast formation by secreting various pro-inflammatory cytokines, including IL-1, IL-17, IL-22, and TNF-alpha [61].

Although hub genes have demonstrated strong correlations with numerous immune cells, further research is needed to explore how these genes influence the pathogenesis of osteoporosis through a series of interactions within inflammatory mechanisms.

Cancer is a major public health issue worldwide and also is a major cause of death in both China and developed countries. Since 2000, there has been a gradual increase in the number of cancer cases, deaths, incidence, and mortality rates in China. Age is a significant risk factor for cancer development. With increased age, the incidence of most malignant tumors significantly escalates [62,63]. Numerous studies have shown that the prevalence of both osteoporosis and chronic kidney disease also increases with age [64,65]. Numerous studies have demonstrated a link between osteoporosis and chronic kidney disease (CKD) with various types of cancer. Extensive cohort studies have confirmed that patients with osteoporosis and CKD face an increased risk of developing cancer [66–69]. Therefore, we selected the hub gene (FAM184A) with the best diagnostic efficacy and conducted a pan-cancer analysis to explore the potential of FAM184A for early diagnosis and targeted therapy in various cancers. In our study, we observed a significant downregulation of FAM184A expression in 22 tumor types compared to normal tissues. Additionally, according to OS and KM curves, we found SARC SKCM and PAAD would have better prognosis with high FAM184A expression. However, studies elucidating the role of FAM184A in the pathogenesis of these cancers are comparatively few. The function of FAM184A may differ among various cancer types, necessitating additional experimental and clinical research to elucidate the specific mechanisms of FAM184A in different tumor contexts, including its impact on cellular behavior, signaling pathways, and interactions with the tumor microenvironment.

There are also some limitations in this study. Firstly, the sample sizes, ages, and genders of the OP and CKD patients were not taken into careful consideration, as the patient data were downloaded from a public database. In addition, as the focus of this study was on peripheral blood mononuclear cells, the subsequent validation experiments could only be conducted using blood samples, which imposed certain constraints on the experimental design. Therefore, validation of the key findings in this study was performed solely using RT–qPCR analysis. Finally, although this study has elucidated the relationship between the expression levels of hub genes, immune cells, and tumor tissues, further molecular biology experiments and clinical trials are required to explore the mechanisms of action of these hub genes.

## 5. Conclusion

Currently, the prevalence of OP patients with CKD remains persistently high, but the molecular association mechanisms between the two diseases have not been fully elucidated. Existing mechanisms related to their pathogenesis remain highly controversial, which directly results in a lack of effective breakthroughs in clinical treatment. Therefore, it is imperative to further explore the common hub genes and core molecular mechanisms of CKD and OP, in order to provide support for the future development of specific targeted drugs. In this study, four hub genes were identified through bioinformatics analysis and machine learning algorithm, and all of them showed excellent diagnostic value in osteoporosis and chronic kidney disease. Additionally, we also explored the relationship between these hub genes and inflammation response to reveal the pathogenetic mechanisms connecting chronic kidney disease and osteoporosis. Lastly, we conducted pan-cancer analysis to explore the relationship between FAM184A and different cancers. These hub genes may offer potential therapeutic targets for CKD, OP, and cancers.

## Supporting information

S1 File Research design flowchart diagram that represents the logical sequence of the analytical steps of this study. (DOC)

S2 File Expression matrix of GSE7158. (CSV)

S3 File Expression matrix of GSE15072. (CSV)

S4 File Expression matrix of GSE56815. (CSV)

S5 File Expression matrix of GSE70528. (CSV)

S6 File RAW data. (ZIP)
