# Supplementary material for: Identification of potential biomarkers for osteoporosis and chronic kidney disease through bioinformatics and machine learning algorithm
Source: PLoS One. 2026 May 4;21(5):e0348515. doi: 10.1371/journal.pone.0348515 (PMC13138668; doi:10.1371/journal.pone.0348515)
Supplement: S1 File — (DOC) [file pone.0348515.s001.doc]

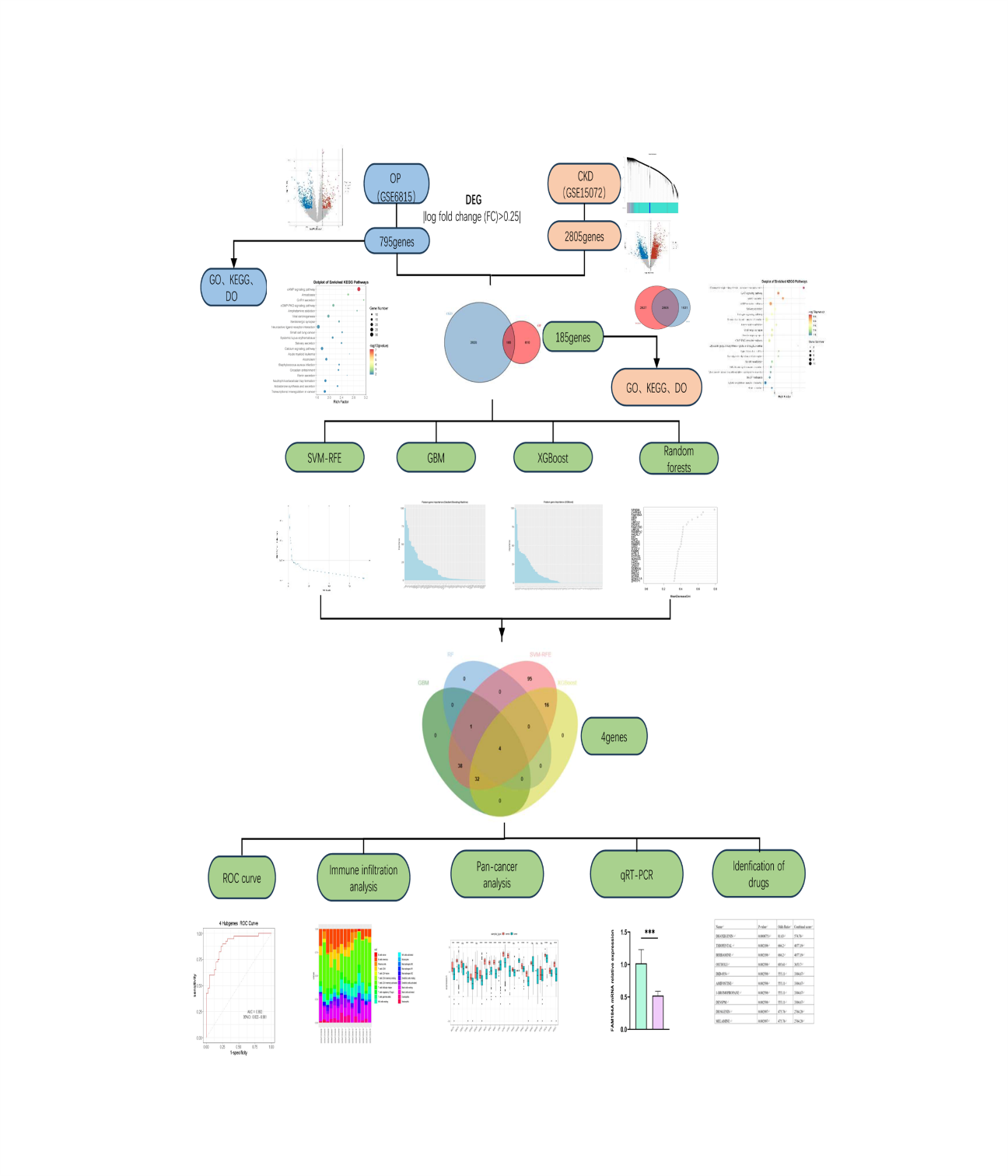


**SI Fig.1 Research design flowchart diagram that represents the logical sequence of the analytical steps of this study**
